# Effects of school-based occupational therapy program for children with disabilities in elementary school in Korea: a case study

**DOI:** 10.1186/s40359-024-01520-3

**Published:** 2024-01-16

**Authors:** Eun-Hwa Jeong

**Affiliations:** Dept. of Occupational Therapy, College of Health Science, Far East University, 76-32, Daehak-gil, Gamgok-myeon, Eumseong-gun, Chungcheongbuk-do 27601 Republic of Korea

**Keywords:** Attention, School adaptation, School-based occupational therapy, Sensory processing, Sensory integration

## Abstract

**Background:**

The purpose of this case study was to explore the effects of a school-based occupational therapy on children’s attention, school adaptation, sensory processing, and motor function for children in special classes in elementary school in Korea.

**Case presentation:**

The subjects of this study were a 7-year-old boy with autism spectrum disorder and a 9-year-old girl with intellectual disability. The school-based occupational therapy program consisted of 10 sessions and was conducted once a week for an hour and a half. The program consisted of classroom activities, use of school facilities, emotional management, and activities based on sensory integration, and was conducted as individual and group programs according to sessions. As a result of the study, all improved when the pre- and post-scores of the two children’s attention assessment, school adjustment scale, sensory processing evaluation tool for the children in school and BOT-2-SF were compared.

**Conclusions:**

Although the results from two cases cannot be generalized, the findings suggest the school-based occupational therapy program may help a positive effect on the school life of children with disabilities. Further investigation is necessary.

## Introduction

It is difficult for children with disabilities to properly perform learning activities, school rules, and social interactions due to physical difficulties, difficulty in controlling emotions, impulsivity, and poor attention in school life [1]. Also, when accompanied by sensory processing disorder, difficulty in following instructions, learning, and controlling behavior can cause school adaptation problems [2].

Occupational therapists prepare students for and participate in important learning and developmental activities within the school environment through school-based occupational therapy [3]. Occupational therapists can help children with disabilities prepare for learning and carry out other related activities in schools. It provides academic and non-academic interventions, including social skills, academic and behavior management, and athletic participation [4].

It has been reported that 90% of occupational therapists in school-based occupational therapy practices perform sensory integration interventions based on ‘Ayres’ theory [4]. Sensory integration is a way to help children improve their adaptive ability to interact with their environment. Therefore, the fundamental purpose is to prevent non-adaptive behaviors or difficulties and improve the quality of life and performance by appropriately interacting with the environment and the body [2–4]. However, when applying these sensory integration interventions to school-based occupational therapy, it is necessary to appropriately apply other intervention for improving school adaptation according to needs. And it is more reasonable to establish an intervention plan based on this after identifying students’ performance skills and non-adaptive behaviors [4, 5].

School occupational therapists in Korea mainly provide intervention based on the medical model, focusing on improving children’s occupational performance problems by improving deficits in subcomponents such as cognitive, motor, and sensory. In previous studies, it was found that writing for learning skills, fine motor and visual motor integration were most often implemented as interventions of school-based occupational therapy [6]. However, most children with school adjustment problems need interventions for overall school performance, such as following rules and instructions, paying attention, and interacting with peers, rather than academic skills [7]. In other words, school-based occupational therapy requires occupational therapists to deal with non-adaptive behaviors that hinder children’s adaptation to school in the natural environment of the classroom. It can potentially lead to a positive impact on academic performance [4–6]. In developed countries where school based occupational therapy was implemented before Korea, school occupational therapists emphasize that therapy services should be provided in natural environments such as classrooms, playgrounds, cafeterias, and hallways in order to achieve academic and functional goals [8].

The role of occupational therapists in schools and educational systems is to facilitate students’ ability to perform tasks or perform meaningful and purposeful activities as students. Therefore, it is necessary for occupational therapists to cooperate with teachers to evaluate students’ functional performance problems in the classroom and to provide necessary interventions to students by discussing effective programs. In this respect, this case study was to confirm the effectiveness of a school-based occupational therapy program that applied a client-centered approach. Therefore, the purpose of this study was to explore the effects of a school-based occupational therapy intervention focusing on school adaptation on children’s the attention, school adaptation, sensory processing, and motor function for children in special classes in elementary school in Korea.

## Case presentation

### Participants

The subjects of this study were two children from a special class at Elementary School in Korea. Subject A was a 7-year-old male with autism, and subject B was a 9-year-old female with intellectual disability accompanied by autistic features. Subject A did not have social interaction including eye contact, frequently stared into space, and had a very short attention span. There were problems in that they often left their seats during class, showed stereotyped behaviors such as going around the classroom in a circle, and frequently made meaningless sounds such as shouting during class. In addition, he was unable to hold a pencil for writing and was unable to use a spoon for eating, so he had to eat with assistance. Subject B often expresses crying and anger when she is not allowed to do what he wants, and often leaves her seat during activities. These characteristics caused problems in peer relationships due to difficulties in controlling and expressing emotions during school life. In addition, problems with social interaction often occurred due to difficulties in conversations appropriate to the situation.

### Procedure

This study was designed to conduct pre- and post-tests on attention, motor function, school adaptation, and sensory processing function of each subject to confirm the effectiveness of the school-based occupational therapy program. This study used convenience sampling. Therefore, the criteria for selecting subjects were students from special classes at the elementary school where the experiment was conducted, who were able to participate in the study. Subjects in this study did not take any additional treatment or psychopharmacological medications.

The intervention program was implemented for 10 sessions from March to June 2022. During the intervention period, the intervention was conducted every Friday from 2:00 to 3:30 p.m.

The program was planned and executed by an intervention team composed of occupational therapists. The intervention goal for each subject was set by the elementary school special class teacher and the intervention team. The evaluation of attention, school adaptation, and sensory processing function was conducted by special class teachers. And The evaluation of motor function was conducted by the intervention team.

Pre- and post-assessments were conducted in sessions 1 and 10, respectively, and were included in the intervention session. Additionally, the evaluator and intervention provider were the same.

### Intervention

The school-based occupational therapy program of this study was constructed based on the framework of sensory integration theory and the school-based occupational therapy model. There were two students in the group, three occupational therapists and one special education teacher running the sessions. The teacher only participated in goal setting, and direct intervention was conducted by occupational therapists. According to the purpose of each session, an individual program suitable for the intervention goal and a group program in which two children participated were conducted. The goal of this school-based occupational therapy program was to improve school adjustment, including learning activities, school rules, and interactions, by mediating each child’s problems. The intervention goal of subject A was to improve attention, and provide an experience of school assignments during activities for school adaptation. The intervention goal of subject B was to improve school adaptation and self-expression skills through school assignment experiences (Table 1).

**Table 1:** Summary of school-based occupational therapy programs

Session	Subject	Purpose	Program
1	Group	Improving social skills through voluntary participation	Pre-evaluation Observation & Rapport formation -Free play -Greetings and conversations -Physical activity
2	Group	School assignment experience and skill improvement	Handicraft activity -Holding a pencil, Writing, Drawing -Self-expression -Follow the rules & instructions
3	Group	Improve peer interaction Improve body function Adjusting to school facilities	Outdoor activity -Follow the rules & instructions -Picking up leaves and twigs as directed -Sticking leaves and branches together on paper -Gluing, Coloring, Writing -Self-expression
4	Subject A	Improve attention	Activity (using Balls, Boards)
	Subject B	Understanding Your Emotions	Emotional card activity
5	Subject A	Improve attention Training in the use of eating utensils	Activity (using Spoons, Clays, Wooden blocks, Water)
	Subject B	Understanding Your Emotions	Emotional card activity
6	Group	Improving sensory processing function Adjusting to school facilities Occupation participation	Activity: Auditorium - Playground - Cafeteria -Complete missions in each location of the school -Stretching, Slide, Swing, Bowling -Receive snacks on trays in the lunchroom -Follow the rules & instructions
7	Group	Improving sensory processing function Improve attention Adjusting to school facilities Occupation participation	Activities: English Class - Health Room - Library -Complete missions in each location of the school -Stretching, Slide, Swing, Bowling -Making a Halloween candy bag (gluing, pasting) -Social interaction (receive candy from the school infirmary)
8	Group	Improving sensory processing function Improve performance pattern (motor, process, communication) Occupation participation	Activity (using Balls, landing nets, steppingstones, baskets, swings)
9	Group	Improving sensory processing function Improve performance pattern (motor, process, communication) Occupation participation	Gross motor activity -Hand-foot coordination, Balance, Proprioception stimulation Handicraft activity -Coloring, Fine motor function
10	Group	Post-evaluation Improve voluntary participation	Post-evaluation Free play

### Assessments

#### Attention assessment

In this study, sub-items of the Korea-Child Behavior Checklist (K-CBCL) were used to evaluate the subject’s attention. K-CBCL is a standardized child and adolescent behavior assessment tool that translated the Child Behavior Checklist developed by Achenbach and Edelbrock (1983) into Korean [9–11]. The Cronbach alpha value of K-CBCL was 0.62-0.86. It is evaluated for children between the ages of 4 and 17, and is divided into a social ability scale and a problem behavior syndrome scale. The social ability scale consists of 13 items of 3 categories (sociality, academic performance, and total social ability). The problem behavior syndrome scale consists of 117 questions in 13 categories (deterioration, physical symptoms, anxiety/depression, social immaturity, thinking problems, attention problems, delinquency, aggression, internalization problems, externalization problems, total problem behavior, sexual problems, and emotional instability). Each item is on a 3-point scale (0 points; never, 1 point; occasionally or infrequently, 2 points; frequent or severe), with a score ranging from 0 to 234 points. Scoring and interpretation of results can be done by creating a profile to ensure that it falls within the clinical range [10, 11]. In this study, 11 items of attention problem among the subscales of the Problem Behavior Syndrome scale were evaluated to evaluate children’s attention. It is interpreted that the higher the score, the lower the attention.

#### School adjustment scale

The school life adjustment scale consists of 4 areas: class attitude, friendship, positive personal behavior, and school rules, with a total of 20 questions [12]. The scale was a 4-point Likert scale of not at all (1 point), a little not (2 points), a little bit yes (3 points), and very yes (4 points). In this study, a total of 10 questions were evaluated by reorganizing them into questions suitable for school life adjustment of children in special classes. It is interpreted that the higher the score, the higher the adaptation to school life.

#### Sensory processing assessment tool for schools

A sensory processing assessment tool for schools was developed to evaluate behaviors related to sensory processing difficulties in school life of school-age children [13]. The evaluation items consisted of 42 items in the general learning activity area, and the detailed areas consisted of tactile processing, movement processing, visual processing, auditory processing, olfactory processing, and multisensory processing. In addition, the arts and sports activity area consist of 15 questions, and the meal time and break time activity area consists of 21 questions, totaling 78 questions [13]. The scale consists of three-point scales of 1 (not so), 2 (normal), and 3 (very so). The score can be calculated by summing the total score for each area and the total score. A higher score indicates difficulty in sensory processing.

#### Bruininks-Oseretsky test of motor proficiency-2-SF

The Bruininks-Oseretsky Test of Motor Proficiency-2-SF (BOT-2-SF) was used to measure children’s motor skills. In its short form, the assessment includes 14 items from eight subtests, reflecting different motor domains: (a) fine motor precision(drawing lines through crooked paths, folding papers), (b) fine motor integration(copying a square, copying a star), (c) manual dexterity(transferring pennies), (d) bilateral coordination(jumping in place—same sides synchronized, tapping feet and fingers—same sides synchronized), (e) balance(walking forward on a line, standing on one leg on a balance beam), (f) speed and agility(stationary hops), (g) upper-limb coordination(dropping and catching a ball with both hands, dribbling a ball with alternating hands), (h) strength(knee push‐ups, sit ups) [14]. The raw score of each item was converted according to the inspection manual, and the total score was obtained by adding the scores of these items.

### Analysis

According to the characteristics of this study, the pre- and post-change values ​​of each subject were presented in a table and graphed. Attention assessment and school life adjustment scale were compared to check changes in variables related to each subject’s list of problems by comparing changes in all items. The analysis of the sensory processing evaluation for school aimed to identify the intervention effect on detailed factors by confirming the change in each area and the total score. BOT-2-SF compared the change in conversion score and total score for each item of the subject.

### Results

The changes in the pre- and post-scores of each subject’s attentions, school adaptation, sensory processing, and motor function are shown in the Table 2. There was a positive change in scores for attention, school adaptation, and sensory processing of all subjects. Subject A was not performed in the BOT-2-SF because He had difficulty following instructions due to his symptoms. Subject B had a positive score change in the BOT-2-SF.

**Table 2:** Summary of study results

	Subject A		Subject B	
	pre	post	pre	post
Attention	19	13	12	8
Adaptation	16	24	26	30
Sensory Processing	161	127	111	97
BOT-2-SF	NA	NA	9	14

**Fig. 1:**
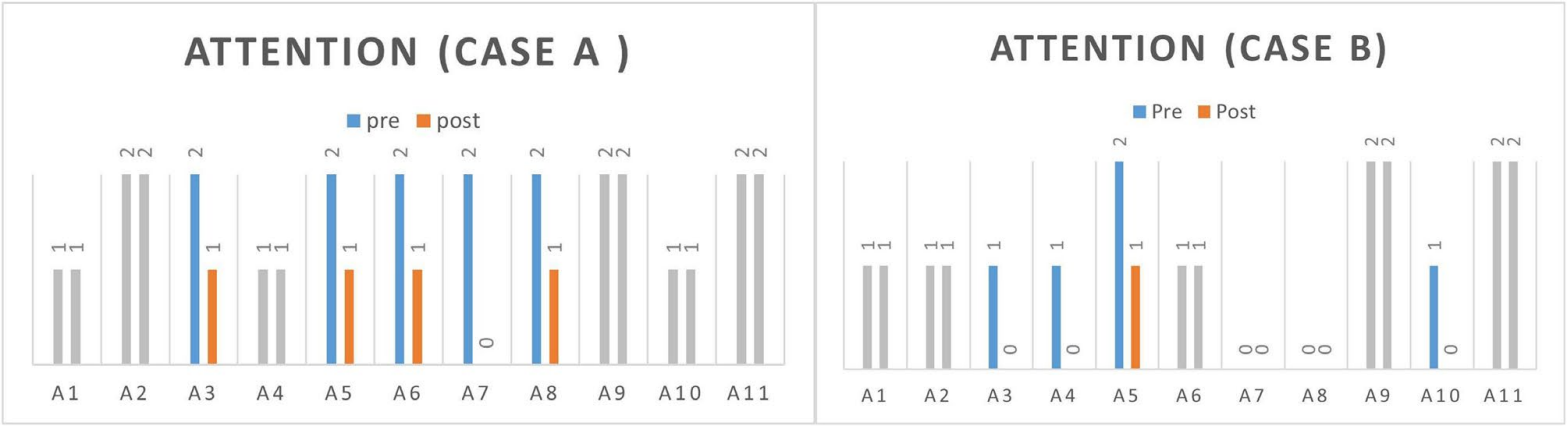
Results of attention assessment A1: acting younger than one’s age, A2: attention problems, A3: hypercactivity, A4: stupor, A5: maladaptive daydreaming, A6: impulsiveness, A7: tension, A8: anxious gestures, A9: poor schoolwork, A10: poor motor function, A11: stare blankly into space Gray means no change in pre-post scores

The change in scores for all items of each subject’s attention assessment is shown in Fig. 1. Subject A had positive score changes in the items of hyperactivity, maladaptive daydreaming, impulsiveness, tension, and anxious gestures. Subject B had positive score changes in hyperactivity, stupor, maladaptive daydreaming, and poor motor function.

**Fig. 2:**
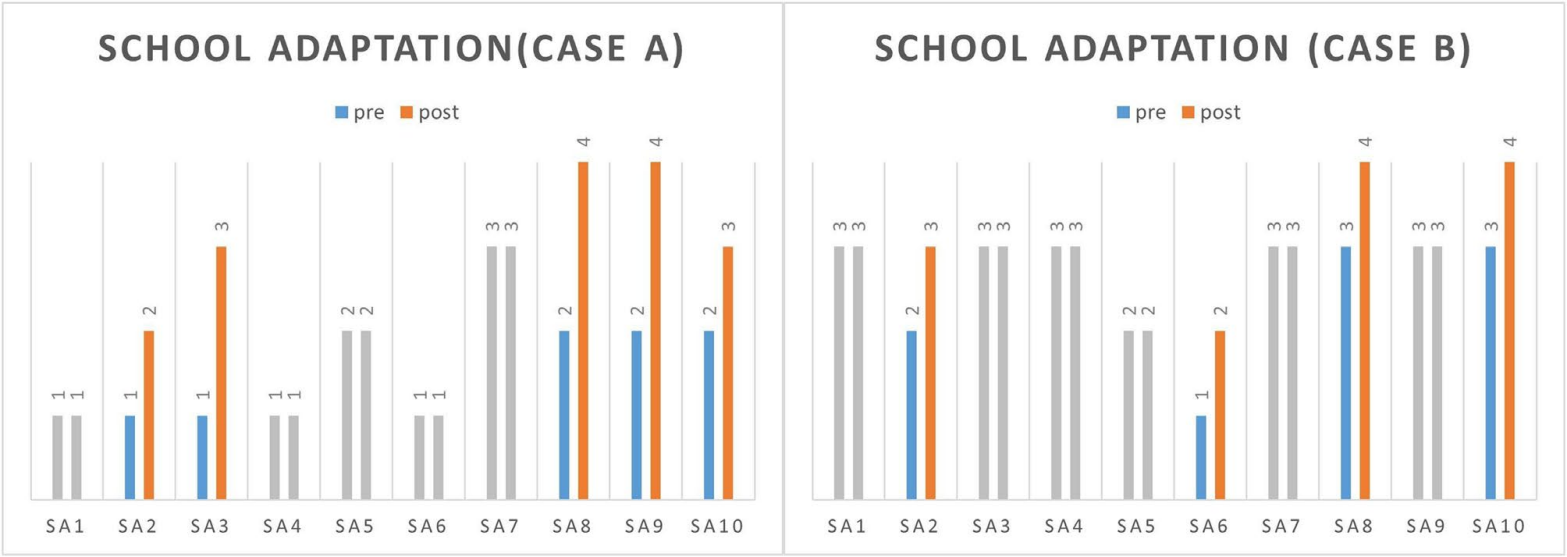
Results of school adjustment scale SA1: The student listens attentively to the teacher’s assignment instructions or explanations. SA2: The student has relationships with several friends. SA3: The student has appropriated physical contact with peers. SA4: The student responds appropriately to praise, blame, and punishment. SA5: The student induces the other person’s interest in an appropriate way. SA6: The student is good at controlling his mood. SA7: The student keeps their school hours and class hours well. SA8: The student uses school facilities carefully. SA9: The student is orderly when using the bathroom. SA10: The student greets the teacher well. Gray means no change in pre-post scores

The change in scores for all items of each subject’s school life adjustment scale is shown in Fig. 2. Subject A had positive score changes in the following items: ‘The student has relationships with several friends.’, ‘The student has appropriated physical contact with peers.’, ‘The student uses school facilities carefully.’, ‘The student is orderly when using the bathroom.’, ‘The student greets the teacher well.’ Subject B had positive score changes in the following items: ‘The student has relationships with several friends.’, ‘The student induces the other person’s interest in an appropriate way.’, ‘The student is good at controlling his mood.’, ‘The student uses school facilities carefully.’, ‘The student greets the teacher well.’

The results of each subject’s sensory assessment tool for schools are shown in Table 3. According to the total score, there was a positive score change for sensory processing in both subjects. Negative score changes appeared in the arts and sports activity area of ​​subject A, but positive changes in the pre- and post-scores of the two subjects were found in all other evaluation items.

**Table 3:** Results of school sensory assessment tool for schools

	Subject A		Subject B	
	pre	post	pre	post
Total score of General learning activities	83	68	61	52
Tactile processing	11	12	10	11
Motor processing	28	22	19	15
Visual processing	25	17	17	12
Auditory processing	8	7	7	5
Olfactory and multisensory processing	11	10	8	9
Arts and sports activities	27	30	24	20
Meal and break-time activities	44	29	26	25
Total score	161	127	111	97

## Discussion and conclusion

This study explored the effects on children’s attention, school adaptation, sensory processing, and motor function through a school-based occupational therapy program consisting of classroom activities, use of school facilities, emotional management, and activities based on sensory integration. The concept of school adjustment is understood as a complex concept consisting of several sub-variables. In summarizing various studies, learning activities, school rules, peer relationships, and teacher relationships are classified as sub-variables [15–17]. In this study, a school-based occupational therapy program aimed at school adaptation was constructed, and the occupational therapy team and the special class teacher cooperated to identify each child’s problems. And it was composed of individual and group programs according to the session.

As a result of this study, both children showed positive change in attention. Both children showed improvement in hyperactivity and maladaptive daydreaming items. This is influenced by handicrafts and physical activities aimed at improving attention, based on previous studies that sensory integration-based activities can cause self-regulation and attention improvement [18–20]. Subject A showed a decrease in scores in the items of impulsivity, tension, and anxious gestures. ASD children like subject A are known to show high correlations with lack of sociability, negative emotions, and anxiety [20]. These factors manifest in the form of self-injurious behavior, aggression, and lack of self-control that negatively affect school life. Therefore, the results of this study can be supported based on previous studies that the process of regulating and processing senses in children with autism can affect the regulation of behavior and emotions [20, 21] In addition, it is thought that not only sensory integration-based programs but also programs implemented for school assignment experience and adaptation to school facilities improved adaptability to activities and places. Subject B showed a positive score change in motor dullness in the attention assessment. Also, subject B showed improvement in the BOT-SF score. These results are considered to have been helpful through the group session applied physical activity, gross motor activity, and sensory integration-based physical activity.

Both children showed positive results on the school adjustment scale. After the intervention, both subjects had relationships with various friends, showed a tendency to use school facilities carefully, and greeted teachers better. In particular, subject A came to have proper physical contact with friends and was found to keep order better when using the bathroom. On the other hand, subject B showed improved emotional regulation. It may be due to the method in which this program identified problems of each subject through a client-centered approach and operated in individual and group sessions. These results are considered to have been induced by the effect of intervention programs aimed at adaptive behaviors for school life, such as school rules, social skills, and use of school facilities, rather than specific academic skills.

The total score of the two children’s sensory processing assessment tool for schools showed a positive change, but there were some differences in the scores of the detailed items. Subject A had relatively improved eye contact during classroom interaction, attention to visual stimuli, and recognition of visual stimuli. In addition, performance improved when lifting and moving the food tray, food or drink did not spill easily while eating, and hyperactivity decreased during breaks. Therefore, it was confirmed that the adaptation behavior in school life was improved. These results can be inferred that various sensory stimuli through fine and gross motor activities influenced visual processing and attention [22]. In addition, it is thought that the meal utensil training and school facility use program influenced the improvement of subject A’s adaptive behavior during meal and break time activities. Subject B showed some improvement in the movement processing score due to reduction in hyperactivity during class activities, improvement in postural control, improvement in both-handed task performance, and improvement in pencil grip. In addition, it showed improvement in visual processing scores by improve attention on visual stimuli and improved spatial arrangement organization during writing. And there was improvement in music and physical activity. These results are supported by previous studies that in the process of receiving and processing various sensory stimuli, motor function, self-regulation ability, and concentration are improved, which affects the performance of activities, cognitive function, and communication function [18–22].

Children with disabilities in special classes have different characteristics for each disease, and there are differences in school adaptation according to age, school type, and severity. Therefore, it can be considered to operate a school-based occupational therapy program by grouping and operating according to the child’s characteristics, age, or intervention goal, or dividing individual and group sessions as in this study. However, in Korea, occupational therapists often provide treatment support services in treatment rooms based on existing medical models rather than supporting students’ school life in a cooperative team with teachers in a school environment. In order for children to adapt to school, it is necessary for teachers to focus on the child’s educational aspects and for occupational therapists to cooperate based on the goal of improving children’s occupational skills [23]. In this regard, active efforts are needed for school occupational therapy through the development of educational programs and establishment of systems to enable interdisciplinary cooperation.

This study conducted a single group program as a case study, and it is very limited to generalize. Additionally, the effects of exogenous variables such as children’s maturation and development cannot be ruled out regarding the results of this study. Therefore, there is a need to expand randomized controlled trials using the school-based occupational therapy intervention program applied in this study. It could be a school-based occupational therapy program targeting all children in special classes or a group of children with a diagnosis different from the subjects of this study. There is also a need to standardize the procedures of school-based occupational therapy programs by organizing the process of evaluation-intervention-outcome and developing a protocol in which this process is progressively repeated.

Occupational therapists working at school need to cooperate with teachers to evaluate students’ functional performance problems in school and provide necessary interventions for students’ school adjustment. At this time, it can be effective that combined intervention of individual and group programs under the intervention goal of school adjustment. In addition, activities based on sensory integration can be helpful to improve non-adaptive behaviors such as hyperactivity, impulsivity, and reduced attention in children with disabilities. It is hoped that this study will emphasize the need for occupational therapy services in the educational field, thereby building a system for collaboration with teachers and demonstrating the expertise of occupational therapists in the school life of children with disabilities.

## Data Availability

The data that support the findings of this study are available from the corresponding author upon reasonable request.
